# Poly-phosphocholination of liposomes leads to highly-extended retention time in mice joints†

**DOI:** 10.1039/d1tb02346b

**Published:** 2022-01-21

**Authors:** Weifeng Lin, Ronit Goldberg, Jacob Klein

**Affiliations:** Department of Molecular Chemistry and Materials Science, Weizmann Institute of Science Rehovot 76100 Israel jacob.klein@weizmann.ac.il

## Abstract

Surface-attached layers of phosphatidylcholine (PC) lipid vesicles (liposomes) may reduce the friction coefficient *μ* (= force-to-slide/load) between the sliding surfaces down to *μ* ≈ 10^−3^–10^−4^ up to tens of atm contact pressures, as high as those in the major joints (hips or knees). Such friction reduction is attributed to hydration lubrication by the highly-hydrated phosphocholine head-groups exposed at the outer vesicle surfaces. It has been suggested therefore that intra-articular (IA) administration of liposomes as potential boundary lubricants may alleviate degenerative, friction-associated joint conditions such as osteoarthritis (OA), which is associated with insufficient lubrication at the articular cartilage surface. To overcome the problem, common to all nanoparticles, of rapid removal by the mononuclear phagocyte system, as well as to ensure long-term colloidal stability during storage, functionalizing liposomes with poly(ethylene glycol) moieties, PEGylation, is often used. Here we describe a different liposome functionalization approach, using poly(2-methacryloyloxyethyl phosphorylcholine), PMPC, moieties (strictly, lipid–PMPC conjugates), and compare the retention time in mice joints of such PMPCylated liposomes with otherwise-identical but PEGylated vesicles following IA administration. We find, using fluorescence labeling and *in vivo* optical imaging, that when PMPC-stabilized liposomes are injected into mice knee joints, there is a massive increase of the vesicles’ retention half-life in the joints of about (4–5)-fold (*ca.* 300–400% increase in retention time) compared with the PEGylated liposomes (and some 100-fold longer than the retention time of intra-articularly injected hyaluronan or HA). Such PMPCylated liposomes are therefore promising candidates as potential long-lived boundary lubricants at the articular cartilage surface, with implication for friction-associated pathologies. Moreover, as lipid vesicles are well known to be efficient drug carriers, such long retention in the joints may enable analgesic or anti-inflammatory agents for joint pathologies to be more efficiently delivered *via* IA administration using PMPCylated liposomal vehicles relative to PEGylated ones.

## Abstract 2

PMPCylated liposomes injected into mice joints show a massive increase in retention half-life compared with PEGylated liposomes (or hyaluronan, HA), making them promising candidates as boundary lubricants at articular cartilage, or as drug carriers.

## Graphical abstract

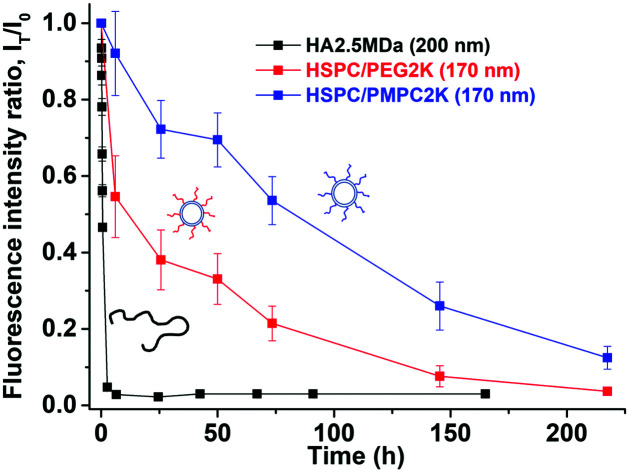

## 1. Introduction

Liposomes have been extensively studied as drug carriers as they can essentially incorporate a wide range of drugs – both hydrophilic and hydrophobic molecules.^1,2^ However, a big disadvantage common to all particulate carriers is that after systemic application, standard liposomes are rapidly removed from the blood, mainly by the cells of the mononuclear phagocyte system, predominantly in the liver and spleen.^3,4^ A major issue, therefore, is molecular engineering of liposomes to increase their retention time.^5,6^ Thus it is known that coating the liposomes with hydrophilic polymers, such as polyethylene glycol (PEG),^7^ zwitterionic polymer,^8,9^ poly[*N*-(2-hydroxypropyl)methacrylamide)] (PHPMA),^10^ and polyvinyl alcohol (PVA),^11^ results in a long blood circulation time and correspondingly-high retention-time of such ‘stealth liposomes’ compared with the pure (unfunctionalized) liposomes. Such PEGylation (regarded as the gold standard for liposome functionalization) is now being widely used for liposomal delivery vehicles, for example in the anticancer medication Doxil®.^12^ It is believed to act through suppression, *via* hydration and steric repulsion, of the adsorption of plasma macromolecules (such as opsonin) onto the vesicles, thereby suppressing their recognition and removal from circulation by the mononuclear phagocyte system.^13–17^

Intra-articular (IA) delivery is another major area where extended retention of liposomal delivery-vehicles is crucial, particularly for the treatment of pathological joint conditions such as osteoarthritis (OA), a painful degenerative disease affecting millions, which is characterized by progressive loss of articular cartilage.^18–21^ Due to the localized nature of OA, IA drug injection is an attractive treatment approach for this disease, which minimizes overall systemic exposure to anti-arthritic drugs.^22^ Meanwhile, a different approach concerning OA therapy deals with the cartilage mechanics either by injection of viscous supplements of hyaluronic acid,^23^ where it is also thought to have lubricating properties,^24^ or by liposome treatment as bio-lubricants.^25^ The idea of using liposomes as efficient boundary lubricants was studied,^26–28^ and it was shown in several studies on model systems that extremely low friction could be attained between boundary layers of PC liposomes, down to friction coefficients as low as *μ* ≈ 10^−4^.^29,30^ This was attributed to hydration lubrication, an emerging paradigm for lubrication in aqueous and biological media,^31,32^ where tenaciously-held yet fluid hydration layers exposed at the outer surfaces of the boundary layers result in a massive reduction of frictional dissipation as the compressed surfaces slide past each other. In the case of PC liposomes, the highly-hydrated phosphocholine groups exposed at their outer surface provide these hydration layers. Formation of strongly-lubricating liposomal boundary-layers was further shown to be highly effective in combination with the cartilage components, including in particular hyaluronan (HA).^33–37^ There is thus a real need for novel IA therapy that will enable long-term liposome retention in joints^38^ both in order to lower the pain or inflammation associated with OA *via* liposomal delivery of analgesic or anti-inflammatory agent, and as a boundary lubricant for articular cartilage.^25^ The use of liposomes for such IA drug delivery or bio-lubricant is of high potential;^39,40^ while large liposomes of size of ∼1 micron seem to be more efficient as IA drug carriers than small vesicles due to their longer retention time within the joint.^41^ These large vesicles (multilamellar vesicles, MLVs) are associated with stability, sterility, and reproducibility problems.^42^ Thus there may be regulatory issues concerning their actual application.^43^ We note in passing that liposomes in the form of large unilamellar vesicles (LUVs) in the size range up to *ca.* 200 nm, prepared by passage through suitable membrane filters (filtering particles of size 0.22 μm or smaller), are of special interest in the context of drug-delivery or boundary-lubricant vehicles.^44^ This is because such liposomes are sterile by virtue of their size and preparation method, and are thus particularly suitable in the context of medical use.^45^

In our previous study,^46^ which was concerned with boundary lubrication in model *in vitro* systems, we created a novel lipid–polymer conjugate where the di-acyl tail of DSPE is attached to a PMPC polymer carrying phosphocholine-like monomers on its backbone. Liposomes incorporated with such conjugates (PMPCylated liposomes) provide excellent stability against aggregation, well comparable with the stability of PEGylated liposomes. Meanwhile, PMPCylated liposomes provide a surface lubricity which is an order of magnitude better than that provided by PEGylated liposomes. Here, in order to develop a novel platform of liposomes for boundary lubricants for cartilage and sustained drug delivery vehicles, we investigate and compare the retention time of PMPCylated liposomes and PEGylated liposomes in the knee joints of mice, using *in vivo* optical imaging, also comparing with the retention time of hyaluronan (hyaluronic acid, HA). The reason for adding this group is that IA injection of HA is a widespread treatment for people suffering from OA as a visco-supplementation.^37^

## 2. Experimental

### 2.1 Materials

Distearoylphosphatidylethanolamine–polyethylene glycol (DSPE–PEG, PEG Mw of 550 and 2000 Da) were purchased from Avanti polar lipids (Alabama, USA). Distearoylphosphatidylethanolamine (DSPE) and hydrogenated soy phosphatidylcholine (HSPC) were purchased from Lipoid (Ludwigshafen, Germany). Triethylamine (99%), 2-(methacryloyloxy)ethyl phosphorylcholine (MPC), copper(i) bromide (CuBr, 99.999%), 2-bromoisobutyryl bromide (BIBB, 98%), *N,N,N*′*,N*′′*,N*′′-pentamethyldiethylenetriamine (PMDETA, 99%), bovine serum albumin (BSA), anhydrous dichloromethane (DCM), and anhydrous chloroform were purchased from Sigma-Aldrich (Israel). DiR (1,1′-dioctadecyl-3,3,3′,3′-tetramethylindotricarbocyanine iodide), DiI (1,1′-Dioctadecyl-3,3,3′,3′-tetramethylindocarbocyanine perchlorate), fetal bovine serum (FBS), and RPMI-1640 medium were purchased from Thermo Fisher Scientific (UK). Hyaluronate-DyLight 755 (2.5 MDa) was purchased from Creative PEG Works (US).

### 2.2 Synthesis and characterization of DSPE–PMPC

DSPE–PMPC was prepared by two steps according to the reported procedure^46^ with minor adjustment (as shown in Scheme S1a, ESI†). The ratio of peaks from the DSPE (*δ* 0.91) and PMPC (*δ* 3.38) (as shown in Scheme S1b, ESI†) determined that the degree of polymerization of DSPE-PMPC is *ca.* 7 (molecular weight of PMPC is *ca.* 2 kDa).

### 2.3 Preparation and characterization of liposomes

Small unilamellar vesicles (SUVs) were made *via* a film hydration–extrusion method. DiR (0.1 mol%), HSPC (97.9 mol%), and DSPE–PMPC (2 mol%) or DSPE–PEG (2 mol%) were dissolved in methanol and chloroform (1 : 1 v/v). Afterwards, the mixture was evaporated under nitrogen overnight, followed by lyophilization overnight to form the final mixed dry powder. MLVs of modified HSPC were prepared in phosphate buffered saline (PBS) by sonication for 15 min at 65 °C, then downsized to form SUVs, ∼80 nm in diameter, at a concentration of 15 mM (by phospholipid concentration), by stepwise extrusion (Northern Lipids Inc., Burnaby, BC, Canada) through polycarbonate membranes starting with 400 nm (5 cycles), 200 nm (8 cycles), and 100 nm (10 cycles).

Large unilamellar vesicles (LUVs) (150 nm) were also made *via* a film hydration–extrusion method. DiR (0.1 mol%) HSPC (97.9 mol%), and DSPE–PMPC (2 mol%) or DSPE–PEG (2 mol%) were dissolved in methanol and chloroform (1 : 1 v/v). Afterwards, the mixture was evaporated under nitrogen overnight, followed by lyophilization overnight to form the final mixed dry powder. MLVs of bare HSPC or stabilized HSPC were prepared in phosphate buffered saline (PBS) by heating for 60 min at 65 °C, then downsized to form LUVs, ∼150 nm in diameter, at a concentration of 15 mM (by phospholipid concentration), by stepwise extrusion (Northern Lipids Inc., Burnaby, BC, Canada) through polycarbonate membranes starting with 400 nm (12 cycles) and 200 nm (12 cycles). Bare HSPC (LUV) formed aggregates with a few hours after preparation. HSPC/PEG or HSPC/PMPC LUVs of 170 nm and 160 nm were separated by centrifugation of prepared LUVs (150 nm) at 15 000 rpm for 90 min and 2 hours, respectively. Liposomes were characterized for size distribution and zeta potential using a Zetasizer Nano-ZS (dynamic light scattering, DLS) instrument (Malvern Instruments Ltd, Malvern, WR, UK). For size distribution measurements, liposomes were diluted with PBS to 1 mM. For zeta potential measurements, liposomes were diluted to 1 mM with PBS, and the samples were measured immediately after dilution. The stability of the liposomes (10 mM) in BSA solution (12 mg mL^−1^) was investigated using DLS.

### 2.4 Intra-articular delivery and retention

All animal experiments were performed according to the guidelines established by the Weizmann Institute of Science, Rehovot, Israel, and approved by the Institutional Animal Care and Use Committee (IACUC). Healthy female CD1 white mice (28–32 g, 8 weeks old, *n* ≥ 5) received either HA or stabilized liposomes to the right joint space. Mice were anesthetized with isoflurane. The hair around the hind limb surgical sites was removed. Either labelled hyaluronic acid or stabilized liposomes were injected into the intra-articular space using a sterile 27-gauge 0.5′′ needle. Then, mice were scanned in an IVIS imaging system at different time points. The excitation and emission detectors were set at 760 nm and 780 nm, respectively.

In order to estimate the safety of using the new PMPC liposomes, a safety study was carried out. In this experiment, mice were IA injected with PMPC liposomes at a 3-fold higher dosage than the concentration being used during the retention studies (30 mM compared with 11 mM). The negative control was a group that was IA injected with saline, and the positive control was a group that was injected with HA. Each group size was 6 mice that were all injected into their right knee joint cavity. At 14 days and 28 days post the IA injections, 3 mice were sacrificed at each time point and were examined by an expert professional histopathologist by both gross pathological evaluation of the tissue and by examination of H&E histology. Each injected knee was compared to both negative and positive controls, relative to the left knee (which was not injected).

### 2.5 Statistical analysis

Statistical analysis was carried out by two-way ANOVA (Analysis of Variance) using GraphPad Prism 7.04 (NS = *p* > 0.05, *p < 0.05, ****p < 0.0001).

## 3. Results and discussion

### 3.1. Liposome stability

**Fig. 1:**
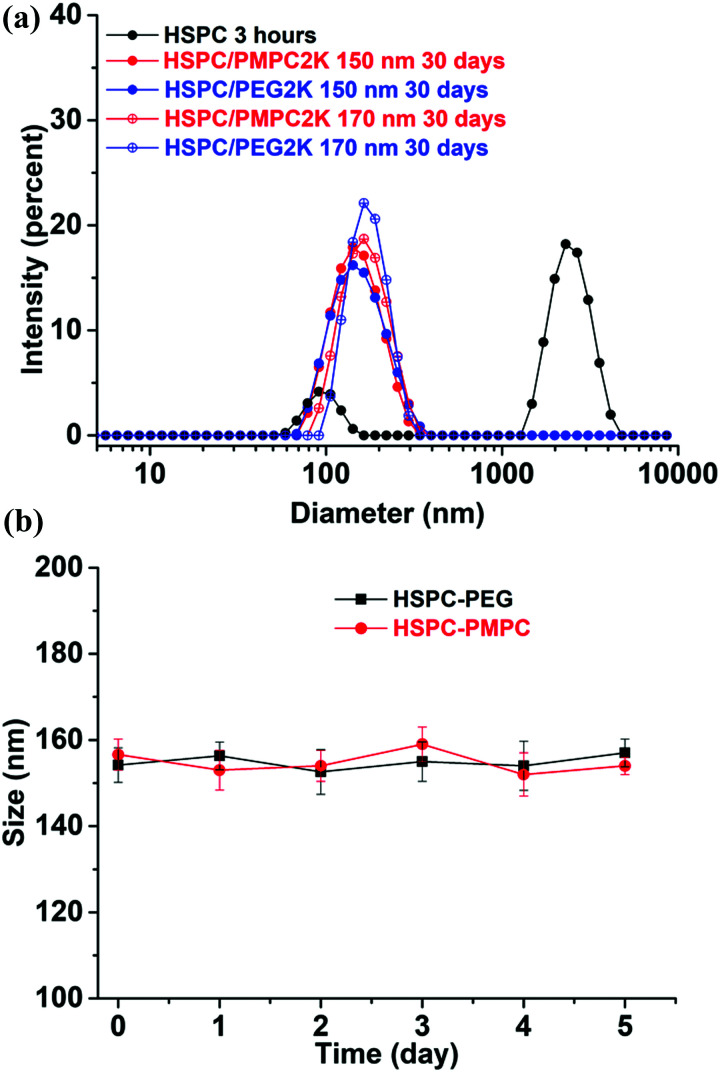
(a) DLS-determined size distribution of HSPC-LUVs, either bare or stabilized by PMPC or by PEG moieties, in PBS at different times following preparation. Measurements were at 1 mM liposome concentration. (b) The stability of liposomes (10 mM) in bovine serum albumin (BSA) solution (1 mg mL^−1^) at 37 °C. The size of the liposome peak (mean ± SD, *n* = 3) is plotted as a function of time.

HSPC is a mixture of 11.4% DPPC (dipalmitoylphosphatidylcholine) and 88.6% DSPC (distearoylphosphatidylcholine). Both DPPC and DSPC are found in cartilage and synovial fluids.^47,48^ Meanwhile, HSPC has been proved to be an excellent boundary lubricant and is widely used for drug-delivery formulations.^49,50^ Therefore, HSPC was selected for our investigation. The DSPE–PMPC conjugate (synthesized by ATRP as described in the methods^46^) and DSPE–PEG conjugate can be incorporated into LUVs of HSPC with the hydrophobic distearoyl tails, forming liposomes stabilized by PMPC and PEG moieties. The stability of liposomes was checked by DLS measurements. As shown in Fig. 1a, 3 hours after the preparation at 4 °C, HSPC SUVs formed aggregates (characterized by a peak at around 2000 nm), whereas no aggregation of HSPC LUVs functionalized with DSPE–PEG or DSPE–PMPC was observed even after one month, indicating that the DSPE–PEG or DSPE–PMPC moieties stabilized the liposome dispersions. Zeta (*ζ*) potential measurements on the stabilized liposomes showed a small negative potential (*ζ*(HSPC-LUV-PEG) = −3.3 ± 0.7 mV and ζ(HSPC-LUV-PMPC) = −4.2 ± 0.9 mV) arising from the negative phosphate groups (–PO_4_^−^) on the DSPE–PMPC or DSPE–PEG conjugates in the physiological salt concentration solution. This result confirms that the PEG or PMPC moieties forms tail which extend as a corona from the liposomes into the surrounding aqueous environment, thereby forming a hydrated steric barrier to aggregation and fusion.

As albumin is the most abundant protein in synovial fluid,^51^ liposome stability study in albumin solution was performed by mixing liposome solutions with a fixed amount of bovine serum albumin (BSA) protein solution of 12 mg ml^−1^ in PBS at 37 °C and monitoring the size by using DLS at different time points. Liposomes prepared from HSPC lipid mixed with 2 mol% of either DSPE–PEG or DSPE–PMPC showed stable solution as there was no change in the measured DLS value during 5 days of incubation time of the liposomes with the BSA solution (as shown in Fig. 1b). These results suggest that the polymer corona added to the liposome surface, either PEG or PMPC prevented interaction of the liposome surface with the BSA, *via* its hydrated steric barrier.^15,52,53^

### 3.2. IA retention time of liposome *in-vivo*

The retention of selected liposomes was studied in our murine model by IA injections of liposomes of chosen size and composition, in the “sterile” size range below 200 nm (see Introduction above). Two types of liposomes were used: PEGylated liposomes, which are the known and most-widely-used state-of-the-art approach for stabilizing liposomes for drug delivery, and the new class of liposomes noted above that has been very recently developed in our laboratory: PC liposomes, stabilized with PMPC moieties. We compared the PEGylated and our novel PMPC-modified liposomes which had otherwise-identical lipid composition and size. As a further control, as noted earlier, we examined a third group consisting of HA polymer. The retention time of unmodified HA within the joint space measured in sheep varies between 12 to 24 h.^54^ Therefore, it is of interest to compare our novel materials to the most common treatment using the IA delivery administration (*i.e.* HA) as well as to the currently-available state-of-the-art stabilized liposomes (PEGylated group) most commonly used for liposomal drug-delivery in general.

**Fig. 2:**
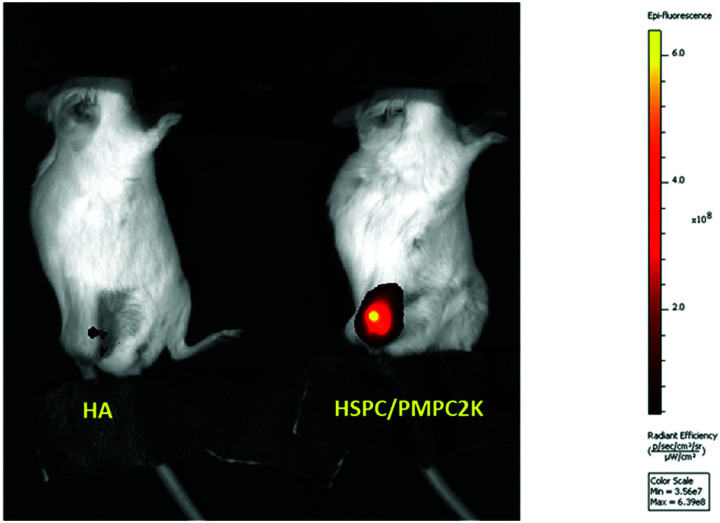
Fluorescence intensity in the knee of mice following 147 hours of IA injection of either fluorescence-labeled HA or HSPC liposomes stabilized with PMPC moieties.

**Fig. 3:**
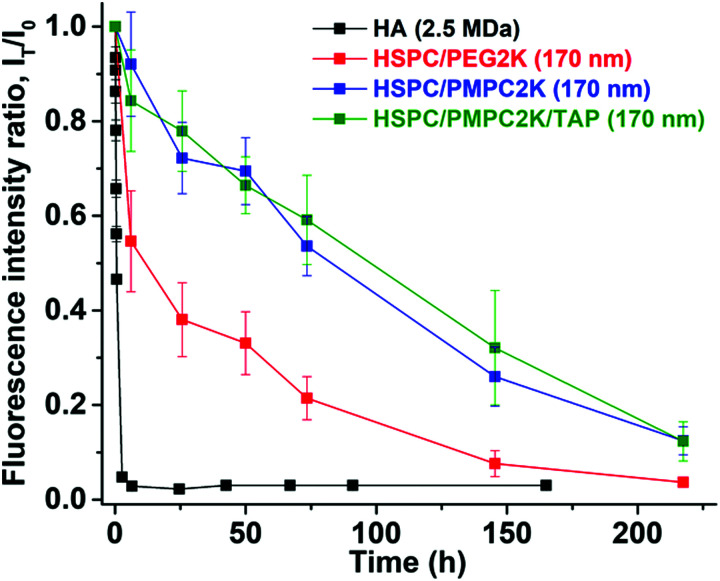
Time decay of fluorescence intensity for the HA and three liposomes systems, showing the much longer *T*_1/2_ of the HSPC/PMPC2K relative to both HA and HSPC/PEG2K. Mean diameter of the liposome vesicles is of *D* = 170 nm.

In our murine model study, several groups (corresponding to HA and various types of liposomes) were used, each with 5–10 mice. Mice received an IA injection of fluorescently-labeled materials (LUV dispersions or HA) that were monitored using a near infra-red camera. All injected substances were functionalized with DiR (1,1′-dioctadecyl-3,3,3′,3′-tetramethylindotricarbocyanine iodide), a lipophilic fluorescent dye, so as to fluoresce at a wavelength of about 755 nm. Results are normalized with respect to the initial signal that was measured immediately following the IA injection, also noted as *T*_0_. A representative example of detection of IA-injected substances in the mice is shown in Fig. 2. Fluorescence intensities as a function of time from injection using such images were used to generate time decay profiles, as shown in Fig. 3. This shows that PMPC-stabilized liposomes of 170 nm in diameter (*M*_PMPC_ = 2000 Da) exhibited a retention half-life *T*_1/2_ of about 85 hours, which was very significantly greater than the half-life of either hyaluronic acid (less than 1 hour) or the half-life of otherwise-identical but PEGylated liposomes with of the same size. As shown in Fig. 3, the half-life of PEG-stabilized liposomes (*M*_PEG_ = 2000 Da) was less than 20 hours. These results indicate that PMPC-stabilization of liposomes enhances retention *in vivo* to a considerably greater extent than PEGylation of liposomes does (*i.e.*, about (4–5)-fold, up to 8-fold in the case of *M*_PEG_ = 550 Da, see below). These results indicate that the strong enhancement of *in vivo* retention of 170 nm liposomes by PMPC (in comparison to PEG) observed herein is not associated with a degree of polymerization of PMPC *vs.* PEG. Results are summarized in Table 1 below. One method to increase the retention of nanoparticles in joint is to utilize electrostatic interactions due to the high density of negatively charged glycosaminoglycans (GAGs) inside cartilage.^56^ Thus liposomes were modified by incorporating 1,2-stearoyl-3-trimethylammonium-propane (chloride salt), which comprises trimethylammonium-propane (TAP) groups that impart a positive charge to the liposome. Liposomes having a diameter of 170 nm were prepared using a 96 : 2 : 2 molar ratio of HSPC to PMPC to TAP (*ζ*(HSPC-LUV-PMPC-TAP) = 0.5 ± 0.3 mV), and their retention following IA injection was evaluated as described above. As shown in Fig. 3, liposomes comprising PMPC and TAP exhibited similar *in vivo* retention properties as those of similarly sized liposomes comprising PMPC without TAP; this might be due to the polymer screening the slightly positive charge or to offset of the positive TAP charge by the negatively-charged DSPE–PMPC moieties. Meanwhile, we noted that liposomes comprising HSPC and TAP without PMPC underwent aggregation after a few days. The reason for not using formulations with higher percentage of TAP is that high cytotoxicity of the positive charged materials has been found.^57,58^

**Table 1:** Summary of the half-life of different liposome and HA systems after IA injection to mice knee joint

Composition	Size (nm)	*T*_1/2_ (hours)
HSPC/PEG2K	∼170	17.2 ± 5.0
HSPC/PEG0.55K	∼170	10.2 ± 3.9
HSPC/PMPC2K	∼170	84.7 ± 5.7
HSPC/PMPC2K/TAP	∼170	89.6 ± 7.8
HA (2.5 MDa)	∼200^55^	<1

Taken together, the above results indicate that PMPC-containing liposomes can be used to enhance strongly the retention of liposome-based drug-delivery vehicles, following IA injection. This may be due to charge–dipole interaction between phosphocholine groups of PMPC and HA in the synovial fluid;^59–61^ this could either improve the sustained release of drugs, in comparison with state-of-the-art drug delivery using PEGylated liposomes, or contribute to better lubrication of articular cartilage in the case where the PMPCylated vesicles attach to its surface.^46^

**Fig. 4:**
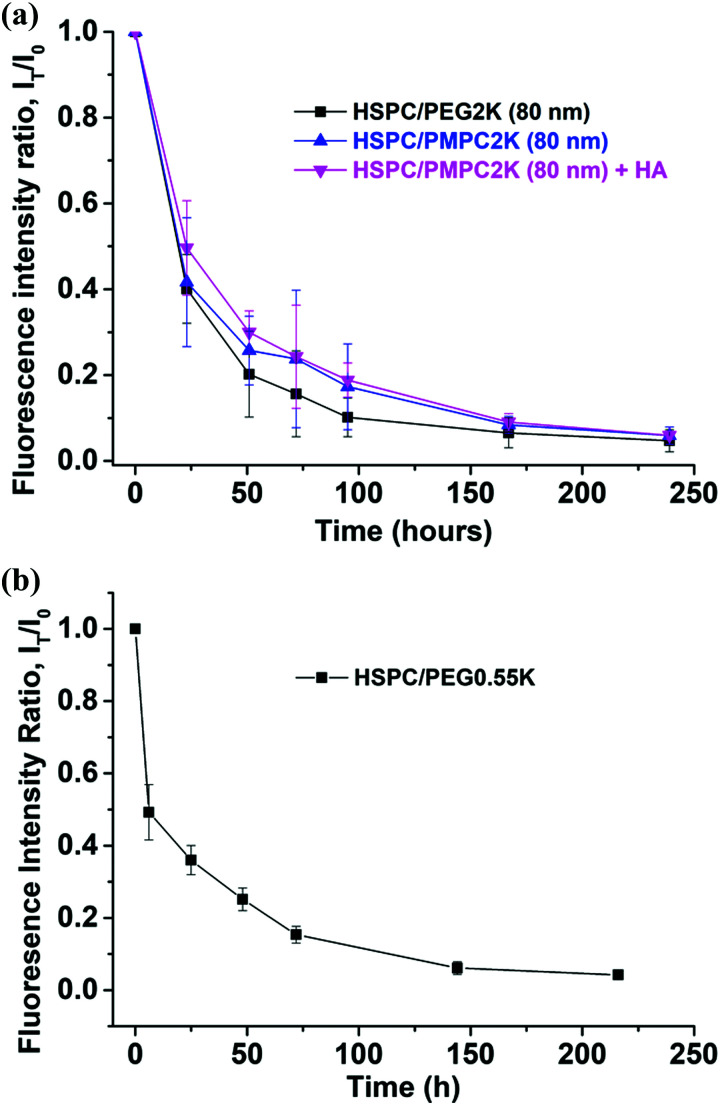
(a) Time decay of fluorescence signals for the HSPC-PEG 0.55 K liposome system, showing a *T*_1/2_ of 10 h. Mean diameter of the liposome vesicles is *D* = 170 nm. (b) Time decay of fluorescence intensity for PEG and PMPC liposomal systems with liposome diameter *D* = 80 nm, showing similar half-lives for such smaller liposomes, even on adding HA.

**Fig. 5:**
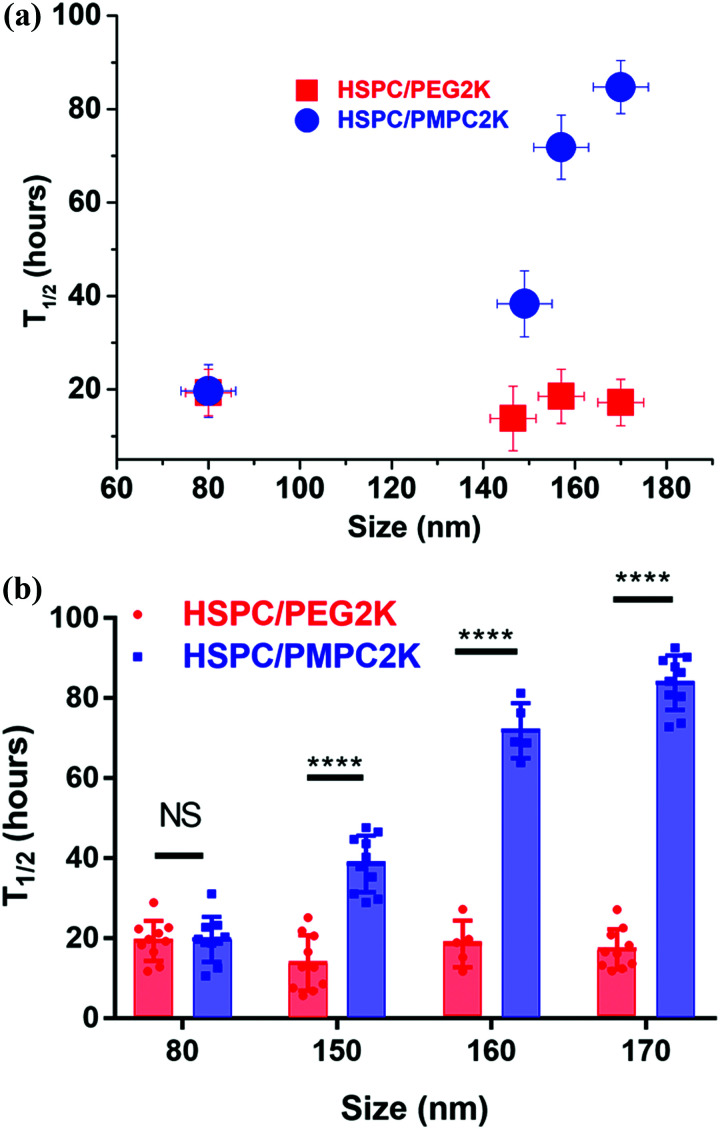
(a) A graph of mean *T*_1/2_ (from several experiments) *versus* the size of the different PMPCylated and PEGylated liposomes used. (b) The full data sets also showing two-way ANOVA analysis (for the PEGylated liposomes there was no significant difference in *T*_1/2_ as a function of their size). Results indicate a highly significant difference between *T*_1/2_ for PEGylated *vs.* PMPCylated liposomes for *D* = 150 nm or larger.

Injecting liposomes of smaller diameters, *D* = 80 nm, lowered dramatically the retention time of the HSPC-PMPC liposomes to a *T*_1/2_ value of 18–22 hours (Fig. 4a), while the HSPC-PEG liposomes showed no correlation between particle size and retention time, as shown in Fig. 5. The above results relate to liposomes stabilized by a polymer (PMPC or PEG) having a molecular weight of 2000 Da. As the MPC monomeric unit has a higher molecular weight than that of ethylene glycol, the total length of the PMPC chain is shorter than a PEG chain of the same molecular weight. In order to assess whether the enhanced retention time associated with PMPC is due to its shorter length, PEGylated liposomes were prepared with a molecular weight of only 550 Da (which has a degree of polymerization which is roughly equal to that of the 2000 Da PMPC). Fig. 4b shows that HSPC-PEG0.55 K liposomes of mean diameter of *D* = 170 nm have *T*_1/2_ ≈ 10 h, which is even shorter than for liposomes functionalized with the longer PEG moieties. Thus this data rules out the possibility that the enhanced PMPC liposome system retention time is due to the shorter PMPC chains compared with the PEGylated liposome system.

There is also an interesting dependence of *T*_1/2_ on liposome diameter, as indicated in Fig. 5. While the retention time of the PEGylated HSPC liposome was not particle size dependent, as PEGylated liposomes of sizes in the range of 80–170 nm in diameter all showed retention time of half-life measured to be in the range of 17 ± 4 hours, PMPC-liposomes showed a rather different trend (Fig. 5). The retention time of PMPC liposomes following IA injection seems to be very sensitive to the particle size as their retention time increases from *ca.* 20 hours to *ca.* 85 hours upon increasing their diameter from 80 nm to 170 nm (Fig. 5). Meanwhile, the safety experiment results (see earlier) indicate that all data for PMPC moieties with different sizes were within normal limits.

## 4. Conclusion

The aim of this study was to examine and evaluate the improved *in vivo* retention time of a new class of liposomes having a PMPC polymer corona. Herein, we demonstrated the ability of the PMPC corona surrounding liposomes to increase their half-life in joints following IA administration, from *T*_1/2_ ≈10–20 hours in case of PEGylation to *T*_1/2_ ≈ 85 hours, a (4–8)-fold increase. This also compares with a *T*_1/2_ of less than 1 hour in case of HA, a commonly used IA-injected visco-supplement. The tendency of the PMPCylated liposome to be retained longer on increasing their diameter, as shown in Fig. 5, is of interest. Surprisingly, in contrast to PEGylated liposomes, the *T*_1/2_ of the PMPCylated ones is sensitive to particle sizes that are smaller than 200 nm. It is possible that the origin for improved retention time for the PMPC-liposomes is its stronger interaction with HA^62^ in the synovial fluid, SF, (arising from charge–dipole attraction) relative to PEG, which being a neutral polymer is less likely to adhere to the negatively-charged HA under physiological conditions. In that case, the size of the PMPC-liposomal complexes with HA, for the larger liposomes (>150 nm) but not for the smaller ones, may exceed the synovial membrane pore size,^63,64^ suppressing escape from the synovial cavity; while for the PEGylated liposomes which do not interact with SF HA, their escape is not suppressed even for *D* > 150 nm. The resulting much-longer *T*_1/2_ for the larger PMPCylated liposomes may better allow them to attach to the articular cartilage surface, thus enabling improved boundary lubrication.^46^ Besides approaches which vary the size,^65^ charge,^66^ and targeting groups^67^ on IA-administered nanoparticles or microparticles to modify their lifetime in the joints, the results of this study suggest a new method to increase the retention time of nano-carriers and nano-lubricants in joints through a novel functionalization route. Any effect of the added liposomes on mechanical properties of the synovial fluid^68^ would clearly also be extended due to this longer retention time. Moreover, liposomes could be designed to have dual functions, both as excellent lubricants for articular cartilage, and at the same time as sustained drug delivery vehicles for treatment of OA. Examples of such drugs could include anti-inflammatory agents.

## Conflicts of interest

The authors have a patent on polyphosphocholinated–lipid conjugates and liposomes stabilized by such conjugates (US10730976B2).

## Supplementary Material

TB-010-D1TB02346B-s001
