# Supplementary material for: Poly-phosphocholination of liposomes leads to highly-extended retention time in mice joints
Source: J Mater Chem B. 2022 Jan 21;10(15):2820–7. doi: 10.1039/d1tb02346b (PMC9007059; doi:10.1039/d1tb02346b)
Supplement: TB-010-D1TB02346B-s001 [file TB-010-D1TB02346B-s001.pdf]

Electronic Supplementary Material (ESI) for Journal of Materials Chemistry B.
This journal is © The Royal Society of Chemistry 2022

Supplementary Information

Poly-phosphocholination of liposomes leads to highly-extended retention time in mice joints

Weifeng Lin, Ronit Goldberg[1], and Jacob Klein*

Department of Materials and Interfaces, Weizmann Institute of Science, Rehovot 76100, Israel

* jacob.klein@weizmann.ac.il

[1] Present address: Liposphere Ltd., Aarava 1, Givat-Shmuel, 5400804, Israel

Synthesis and Characterization of DSPE-PMPC

The ATRP initiator DSPE-2-bromoisobutyrate (DSPE-Br) was prepared as follows: DSPE (4.93 g, 5.6 mmol) was dispersed in dried chloroform (100 mL) with triethylamine (1.4 mL, 11.2 mmol) for 0.5 h at room temperature. BIBB (0.55 mL, 5.6 mmol) was then added dropwise into the mixture. The mixture would form a clear solution gradually, and was stirred for 12 h at 40 °C. The solution was washed with 1 M hydrochloric acid solution (100 mL×3), and pure water (100 mL×3). A white powder was obtained after removing the chloroform using a rotary evaporator (yield: 96%). DSPE-PMPC was synthesized using atom transfer radical polymerization (ATRP). DSPE-Br (1.26 g, 1.4 mmol) was dissolved in DCM (8 mL), and MPC (1.94 g, 6.58 mmol) was dissolved in ethanol (8 mL). The two solutions were mixed in a round-bottomed flask equipped with a septum and a magnetic stirrer. PMDETA (600 μL, 2.8 mmol) was added and the flask was placed in an oil bath at 40 °C, and degassed with a stream of N_2 for 0.5 h. Then, CuBr (210 mg, 1.4 mmol) was added quickly and the mixture was degassed again for 0.5 h. The solution was stirred overnight at 40 °C. Then, the solution was dialyzed (CelluSep membrane having a molecular weight cut-off value of 1000) against ethanol and water for 48 h, respectively. The polymer DSPE-PMPC was obtained after lyophilization (yield: 51%). ^{1}H NMR (Bruker AMX-300 NMR spectrometer, 300 MHz, $CDCl_3/CD_3OD$ 1:1): chemical shift (ppm): 0.53 (3H, $CH_3{-}CR{-}CH_2{-}$), 0.91 (28H, $-(CH_2)_{14}{-}CH_3$), 1.25 (2H, $-CH_2{-}CR{-}CH_3$), 2.94 (9H, $-N^+(CH_3)_3$), 3.39 (2H, $-O{-}CH_2{-}CH_2{-}N^+(CH_3)_3$), 3.70 (2H, $-C{-}O{-}CH_2{-}$), 3.85 (2H, $-CH_2{-}O{-}P$), 3.95 (2H, $-CH_2{-}CH_2{-}N^+(CH_3)_3$).

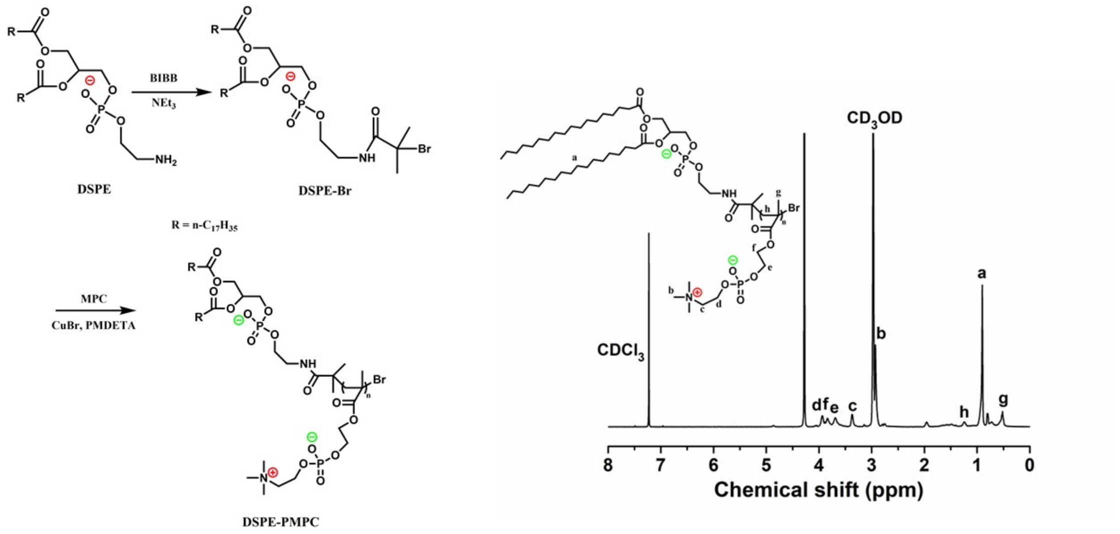

(a) (b)

Scheme S1. (a) Synthesis procedure of the initiator DSPE-Br and lipid-polymer conjugate DSPE-PMPC; (b) ^{1}H NMR characterization of DSPE-PMPC in $CD_3OD/CDCl_3$ (1:1).
